# Did the sweetness of beverages change with the Chilean Food Labeling and Marketing Law? A before and after study

**DOI:** 10.3389/fnut.2022.1043665

**Published:** 2022-10-28

**Authors:** Natalia Rebolledo, Maxime Bercholz, Camila Corvalán, Shu Wen Ng, Lindsey Smith Taillie

**Affiliations:** ^1^Department of Nutrition, Gillings School of Global Public Health, University of North Carolina at Chapel Hill, Chapel Hill, NC, United States; ^2^Carolina Population Center, University of North Carolina at Chapel Hill, Chapel Hill, NC, United States; ^3^Institute of Nutrition and Food Technology (INTA), University of Chile, Santiago, Chile

**Keywords:** sweetness, Food Labeling, front-of-package labels, beverages, Latin America

## Abstract

There has been a rapid proliferation of policies around the globe to reduce sugar consumption, yet there is little understanding as to whether these policies have led to changes in the overall sweetness of products, which is essential for understanding long-term effects on food preferences and intake. For example, the implementation of Chile’s Law on Food Labeling and Advertising led to decreases in the sugar content of non-alcoholic packaged beverages and increases in non-nutritive sweeteners (NNS) use in these beverages. Given that NNS have greater sweetness intensity than sugars, it is unclear what was the net effect of these changes on the sweetness of purchased beverages. Using longitudinal household purchase data (*n* > 2,000 households), we measured the changes in the sweetness of beverage purchases after implementing the first phase of the Chilean law and examine if there were differences by key family sociodemographic variables. We developed three sweetness indices: (1) NNS sweetness, including the sweetness of the six NNS most consumed by Chileans; (2) total sugars sweetness, including the sweetness from total sugars; and (3) total sweetness, combining the sweetness from NNS and sugars. Using fixed-effects models, we compared the observed post-law purchases to a counterfactual based on pre-law trends. We found that NNS sweetness increased relative to the counterfactual, while total sugars sweetness decreased after the law. However, the absolute changes in NNS sweetness were almost entirely offset by the decreases in total sugar sweetness, leading to no change in the total sweetness of beverage purchases. Additionally, there were no differences in the sweetness changes by family sociodemographics. Our findings indicate that Chilean consumers are exposed to similar sweetness levels in their beverages after the law. Future research should explore whether sweetness also remained consistent in dietary intake.

## Introduction

To reduce the intake of sugars, governments have implemented policies such as sugar-sweetened beverages taxes and warning labels (1, 2). However, mounting evidence suggests that these laws may lead to food reformulation, with sugars being substituted with non-nutritive sweeteners (NNS) (3–6). Indeed, there is growing evidence of a global increase of NNS in the food supply (6–8). Nevertheless, there is currently a minimal understanding of how these decreases in sugar and potential increases in NNS interact to affect the overall sweetness of the food supply. Total sweetness may increase because NNSs have a sweetening power that ranges from 100 to 20,000 times greater than sucrose (tabletop sugar), and smaller amounts of NNS are required to produce the same sweetness level (9, 10). However, producers who reformulate likely want to keep taste alterations to a minimum, limiting changes to total sweetness. From a health perspective, there are concerns about how sweetness may modify sweet preferences, particularly in children. In children, the sweetness obtained from sugars or NNS could reinforce sweet preferences that might lead to overconsumption of foods and beverages containing sugars that could persist for a lifetime (11–14).

Data from Chile provide a helpful case study for understanding whether common sugar reduction policies influence overall sweetness. In 2016, the Chilean Ministry of Health implemented the Law of Food Labeling and Advertising (from now on, “the law”). The law requires mandatory front-of-package (FOP) warning labels, restrictions on marketing directed to children under 14 years, and prohibition of school sales of packaged products high in total sugar, saturated fats, sodium, or calories (15, 16). The law provides an excellent opportunity to study changes in sweetness, particularly among beverages. Before the law, beverages were one of the primary sources of total sugars and NNS in the Chilean diet (17–19). After the law, companies reformulated beverages to reduce their sugar content (3), and the proportion of beverages containing NNS increased from 72 to 82.6% (6). Additionally, in preschoolers, the NNS intake increased between 43.8 and 134.6% depending on the NNS type (20), and beverages were the main dietary source of NNS after the law (20, 21). Lastly, beverages were the primary sources of sweetness in a sample of Chilean toddlers (22).

Due to the unique reporting requirements in Chile, which mandate that NNS content be stipulated on packaged foods and beverages, we were able to estimate changes in the quantitative sweetness of beverage purchases by constructing sweetness indices for NNS, total sugars, and total sweetness (NNS combined with total sugars). The objectives of this study were to measure the changes in quantified sweetness in beverage purchases after the implementation of the first phase of the Chilean law and examine if there were differences by key sociodemographic variables.

## Materials and methods

### Study population

Kantar WorldPanel Chile. These longitudinal data include purchases from a panel of over 2,000 households located in urban areas with > 20,000 inhabitants, designed to represent the urban population in Chile. Our analytic sample included 2,380 individual households, with an average follow-up period of 28.4 months, providing 67,646 household-month observations.

To measure household beverage purchases, enumerators visited households weekly to collect information on beverages purchased. Information on each purchase was collected by scanning products’ barcodes using a handheld barcode scanner or using a codebook to assign barcodes for bulk products or other products without barcodes. Interviewers reviewed weekly receipts, conducted household pantry inventories, and checked empty product packages stored in a bin between interviews to ensure products were not double-counted. Data collected on each purchase included volume [milliliters (mL)], barcode, price per unit, retail channel, brand, package size, and date of purchase. Data were aggregated and analyzed at the household-month level. As in previous evaluations of the Chilean law (23, 24), we defined all household beverages purchased between January 1, 2015 and June 30, 2016, as pre-implementation of phase 1 of the Chilean law, and all household beverages purchased collected between July 1, 2016 and December 31, 2017, as post-implementation of phase 1 of the law.

### Ethics

The ethics committee of the Institute of Nutrition and Food Technology of the University of Chile approved this study. This study was exempt from review by the University of North Carolina, Chapel Hill Institutional Review Board because the study used de-identified secondary data.

### The Chilean Food Labeling and Advertising Law

The law requires packaged foods and beverages with added sugar, sodium, or saturated fat and exceeding set thresholds for these nutrients or overall calorie content to carry FOP warning labels with the words “high in” sugar, sodium, saturated fat, and/or calories. The products with FOP warning labels are also subject to child-directed marketing restrictions and banned from sale or promotion in schools and nurseries. The law was implemented in three phases (June 2016, 2018, and 2019), with increasingly restrictive nutrient thresholds (Supplementary Table 1). The sugar threshold for liquids in the first phase was 6 g of sugar per 100 mL of liquid.

### Chilean Nutrition Facts Panel Data

We used Nutrition Facts Panel (NFP) data to develop the sweetness indices. The NFP database contained nutrition information for packaged products available in the Chilean food supply. These data were obtained from photographs of products that a team of Chilean nutrition research assistants collected in stores located in Santiago during the first quarters (January–March) of 2015, 2016, and 2017. The data collection methods have been previously described (25). NFP data were linked to household beverage purchases at the product level and reviewed by a team of nutritionists for accuracy and classification into food groups, using a similar process as in previous household purchase evaluations (23, 24, 26, 27).

We first linked purchases to the 2015 and 2016 NFP data for the pre-law period. For the post-law period, we linked purchases to the 2017 NFP data. Linkages were based on the barcode, brand name, and product description. Of total purchases, 94.10% were linked to collected NFP data. If no collected NFP data were available for a purchased product, it was linked to Mintel Latin America (5.88%) or other NFP data sources (0.02%).

### Beverage group categorization

We categorized each non-alcoholic beverage available in Kantar WorldPanel data into groups used in previous studies (Supplementary Table 2) (23). We excluded formulas for infants, children, and adults.

### Total sugars and non-nutritive sweeteners content of packaged beverages

The NFP data included total sugars and the amounts of eight NNS in packaged beverages because Chile mandates that producers declare the content of acesulfame K, aspartame, cyclamate, saccharin, sucralose, steviol glycosides, alitame, and neotame (28). We excluded alitame and neotame because Chileans do not widely consume them (17, 29, 30), and they are not commonly added into beverages available in the Chilean beverages supply (6, 31).

### Sweetness indices

We developed three sweetness indices to measure how the sweetness of purchases changed after implementing the Chilean policy based on the source of the sweetness: (1) NNS sweetness, including the sweetness of the six NNS mentioned previously; (2) total sugars sweetness, including the sweetness from total sugars; and (3) total sweetness, combining the sweetness from NNS and sugars.

To develop the NNS sweetness index, we converted the milligrams of each NNS into grams because total sugars is reported in grams in the NFP. Then we transformed the purchased grams of each NNS into sucrose equivalents using mean sucrose equivalents (Supplementary Table 3). For the NNSs with a range of sucrose equivalents, we selected the middle value between the range. We created two different sweetness indices for sensitivity analyses, one using the lower limit of sucrose equivalents for NNS and the second using the higher limits. To create the sugar sweetness index, we assumed that total sugar was equivalent in sweetness to sucrose (tabletop sugar). We selected sucrose because the sweetness is measured using reference solutions containing different sucrose concentrations in increasing amounts, and the sweetness of NNS is also measured in sucrose equivalents (10, 32–37). To create the total sweetness index, we combined the sucrose equivalents from NNS purchases with the sucrose equivalents from total sugars purchased. We present an example of the calculation of the sweetness indices for an 8-fl oz (237 mL) soda that contained 28.7 g of sugar, 39.6 mg of acesulfame K, and 14.5 mg of sucralose in Table 1.

**TABLE 1:** Creation of sweetness indices using as example an 8-fl oz (237 mL) soda that contained 28.7 g of sugar, 39.6 mg of acesulfame K, and 14.5 mg of sucralose.

	Grams	Sucrose equivalents	Sweetness
Sugar	28.7	1	28.7
Acesulfame K	0.0396	165	6.5
Aspartame	0.0145	600	8.7
**NNS sweetness**			15.2
**Total sugar sweetness**			28.7
**Total sweetness**			43.9

### Outcomes

Our primary outcomes were NNS sweetness, total sugars sweetness, and total sweetness, all in terms of sucrose equivalent/capita/day. We considered total sweetness as the main outcome in our analyses for the potential effect of the policy on sweetness changes.

### Covariates

The covariates included in the model were based on previous evaluations of Chile’s law (23, 24). We included household characteristics including the head of household’s educational level (less than high school, high school, more than high school), household assets index (developed using factor analysis based on the number of rooms, bathrooms, and cars owned. It was specified as a continuous variable for primary analyses and as a 3-level categorical variable based on tertiles for moderation analyses), and household composition (a set of discrete variables treated as continuous variables, each with the number of people in the following age categories: children 0–1 year, children 2–5 year, children 6–13 year, adolescents 14–18 year, female adults > 18 year, and male adults > 18 year). Monthly region-level unemployment rates from the Chilean National Institute of Statistics were included as contextual measures in the data analysis because trends in economic activity could influence food and beverage purchases (38). We included indicator variables for each calendar month (1–12) to adjust for seasonality and a linear time trend (with monthly intervals).

We added a binary variable for moderation analyses, which indicated if households had children under 14 years old (yes/no). We selected the 14 years cutoff because that is the cutoff age for child-directed marketing restrictions according to the Chilean law.

### Statistical analyses

We conducted all the statistical analyses using Stata 16.1 (College Station, TX, USA), and statistical significance was set at *p* < 0.05. We preregistered the statistical analyses on the Open Science Framework on March 2nd, 2022.^1^

#### Descriptive and unadjusted analyses

We examined the sociodemographic characteristics of households participating in Kantar WorldPanel Chile each year (2015, 2016, and 2017). We also compared the unadjusted mean sweetness of purchases before and after the law, clustering the standard error at the household level. Lastly, we also compared the distributions of the sweetness indices before and after the law. We treated the data as repeated cross-sections and used the Kolmogorov–Smirnov test to compare the distributions before and after the law.

#### Adjusted analyses

We used a quasi-experimental approach for our analyses because the Chilean policy was implemented nationally. That is, the entire population was exposed to the policy simultaneously, preventing a randomized controlled experimental design. We examined the changes in the average sweetness of household beverage purchases before and after policy implementation. Similar to prior evaluations (23, 24), we constructed a counterfactual (a hypothetical scenario) to understand what the post-policy purchases may have looked like if the policy had not been implemented. To do this, our specifications included a binary variable for the policy period (pre vs. post) and its interaction with the linear time trend (to allow for both level and trend changes). We constructed the counterfactuals by predicting purchases in the post-policy period based on pre-policy trends. Consistent with prior evaluations, we included 18 months of data before and after the policy was implemented (23, 24).

We used longitudinal fixed-effects models (-xtreg- in Stata) to estimate the mean changes in the sweetness of beverage purchases. We used our models to compare the non-counterfactual predicted sweetness in the post-policy period to the counterfactual. We estimated the absolute and relative differences between the predicted value and the counterfactual in the post-policy period using the -dxdy- option of Stata’s margins command for all counterfactuals. The 95% confidence intervals and *p*-values for the absolute differences were derived using standard errors obtained by the Delta method.

To test whether policy-related changes in purchases varied by socioeconomic status or household composition, we added an interaction with the educational level of the head of household, household assets, or an indicator variable for the presence of children under 14 years old.

We looked both at differential time trend changes and differential counterfactual differences in the group. For the differential time trend, we analyzed the triple interaction between socioeconomic status or household composition with the policy period and the linear time trend. We also compared the difference between the post-policy predicted and counterfactual mean across levels of education, assets, and households with children under 14 years. We used clustered standard errors at the household level in all adjusted analyses and controlled for the covariates mentioned above.

#### Sensitivity analyses

In our primary analyses, we developed the sweetness indices that used the mean sucrose equivalents for each NNS type. We were concerned about how results would change using the lower and higher sweetness of each NNS reported in the literature. Therefore, we developed additional sweetness indices for the NNS and total sweetness using the lower and higher sweetness values. Then, we repeated our counterfactual analyses.

## Results

### Descriptive and unadjusted analyses

The Kantar WorldPanel Chile sample included 2,076–2,099 households over 2015–2017, representing 22,028–22,826 household-months of observation. Over these 3 years (Table 2), the percentage of households with lower educational levels decreased (from 31 to 27%) while households with higher educational levels increased (from 31 to 34%). We also observed a slight decrease in the mean number of children between 0 and 1 years old. There were no variations in other covariates.

**TABLE 2:** Unweighted sociodemographic characteristics of the Kantar WorldPanel Chile analytic sample, from 2015 to 2017.

Characteristics	2015		2016		2017	
**No. of households**	2,099		2,076		2,098	
**No. of HH-month observations**	22,826		22,792		22,028	
**Head of HH education, n (%)**						
Less than high school	652	(31%)	584	(28%)	575	(27%)
High school	793	(38%)	820	(39%)	814	(39%)
College or greater	654	(31%)	672	(32%)	709	(34%)
**Household assets index, n (%)**						
Low	788	(38%)	750	(36%)	780	(37%)
Middle	635	(30%)	648	(31%)	649	(31%)
High	676	(32%)	678	(33%)	669	(32%)
**Region, n (%)**						
Santiago	915	(44%)	905	(44%)	876	(42%)
Valparaiso	225	(11%)	222	(11%)	258	(12%)
Central South	240	(11%)	243	(12%)	244	(12%)
Bio-Bio	230	(11%)	221	(11%)	238	(11%)
South	248	(12%)	250	(12%)	258	(12%)
North	241	(11%)	235	(11%)	224	(11%)
**Household composition, n (%)**						
Without children < 14 years	869	(41%)	888	(43%)	924	(44%)
With children < 14 years	1,230	(59%)	1,188	(57%)	1,174	(56%)
**Monthly regional unemployment rate, mean (SD)**	6.3	(1.0)	6.5	(1.2)	6.7	(1.1)

In unadjusted analyses, before the law was implemented, the NNS sweetness of beverage purchases was 9.1 sucrose equivalents/capita/day, while the sweetness of total sugars was 19.2 sucrose equivalents/capita/day (Supplementary Table 4). The total sweetness of beverage purchases was 28.3 sucrose equivalents. After the law, the sucrose equivalents increased by 1.2 (*p*-value < 0.01) for NNS sweetness. Total sugars and total sweetness decreased by 4.4 and 3.2 sucrose equivalents/capita/day, respectively.

When looking at changes in the distribution, we observed right shifts in the distribution of the NNS sweetness (Supplementary Table 5). We observed a left shift in the distribution of total sugars and total sweetness.

### Adjusted analyses

**FIGURE 1:**
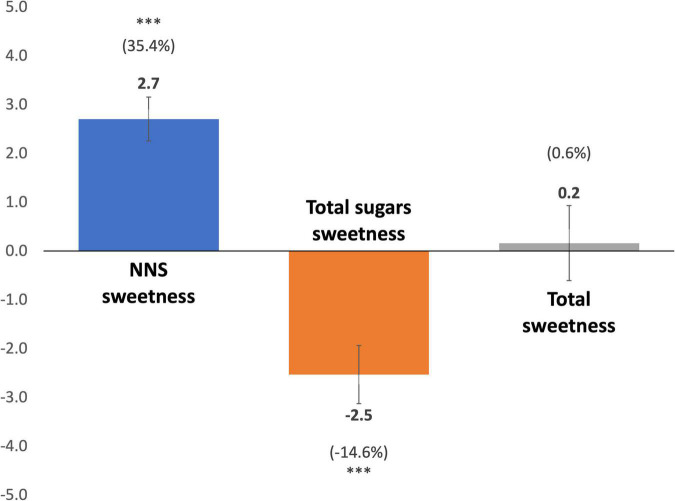
Mean differences in sweetness between estimated adjusted post-policy purchases and estimated adjusted counterfactual post-policy purchases. Estimates are derived from fixed-effects models comparing post-policy sweetness of purchases to counterfactual post-policy sweetness of purchases based on pre-policy trends for each sweetness index. Purchase data provided by Kantar WorldPanel Chile. Error bars indicate 95% confidence intervals. NNS, Non-nutritive sweetener. ***P-value < 0.05.

Compared with the counterfactual, there were no changes in the total sweetness of beverage purchases (Figure 1 and Supplementary Table 6). The sweetness from NNS increased by 2.7 sucrose equivalents/capita/day (95% CI 2.3–3.2, *p* < 0.01) or a relative 35.4% increase, while total sugars sweetness decreased by 2.5 sucrose equivalents/capita/day (95% CI −3.1 to −1.9, *p* < 0.01) or a relative 14.5% decrease.

### Moderation analyses

We presented the differences between the counterfactual and the observed sucrose equivalents in Table 3. The mean counterfactual and the observed sucrose equivalents after the law are shown in Supplementary Table 7. We found no significant interactions between the time trend, the post-policy period, and educational level, household assets, or households with children under 14 years for total, NNS, and total sugars sweetness (Table 3, p-interaction > 0.05). Additionally, the pairwise comparisons between groups were not statistically significant.

**TABLE 3:** Mean differences between the estimated adjusted post-policy sweetness purchased from beverages and estimated adjusted counterfactual post-policy purchases by educational level, household assets, and households with or without children under 14 years.

	Non-nutritive sweetener sweetness (sucrose equivalents)				Sugars sweetness (sucrose equivalents)				Total sweetness (sucrose equivalents)			
	Absolute difference (95% CI)	*P*-value	*P*-interaction	Relative difference	Absolute difference (95% CI)	*P*-value	*P*-interaction	Relative difference	Absolute difference (95% CI)	*P*-value	*P*-interaction	Relative difference
**Education**												
Less than high school	2.8 (2.0, 3.6)	<0.01	0.08	41.6	−2.2 (−3.4, −1.0)	<0.01	0.72	−12.3	0.6 (−0.9, 2.1)	0.44	0.53	2.4
High school	2.4 (1.7, 3.1)	<0.01		32.2	−2.0 (−2.9, −1.1)	<0.01		−11.7	0.4 (−0.7, 1.6)	0.47		1.7
College or greater	3.1 (2.2, 3.9)	<0.01		35.6	−3.3 (−4.3, −2.2)	<0.01		−18.8	−0.2 (−1.6, 1.2)	0.80		−0.7
**Household assets**												
Low	2.4 (1.7, 3.2)	<0.01	0.70	35.4	−2.7 (−3.8, −1.7)	<0.01	0.41	−15.0	−0.3 (−1.6, 1.0)	0.67	0.43	−1.1
Middle	2.6 (1.8, 3.4)	<0.01		35.2	−2.2 (−3.3, −1.2)	<0.01		−13.0	0.4 (−1.0, 1.8)	0.59		1.6
High	3.1 (2.3, 3.9)	<0.01		36.3	−2.3 (−3.4, −1.3)	<0.01		−14.4	0.8 (−0.6, 2.1)	0.25		3.2
**Household with children < 14 years**												
No	3.7 (2.9, 4.5)	<0.01	0.06	41.7	−2.7 (−3.8, −1.6)	<0.01	0.63	−13.8	1.0 (−0.4, 2.4)	0.16	0.47	3.5
Yes	2.1 (1.6, 2.5)	<0.01		31.4	−2.2 (−2.9, −1.5)	<0.01		−14.3	−0.1 (−1.0, 0.7)	0.74		−0.6

Estimates derived from fixed-effects models comparing post-policy sweetness of beverages purchases to counterfactual post-policy sweetness of beverages purchased based on pre-policy trends for each sweetness index. Covariates included head of household education level, household composition, household assets, indicator variable for households with children > 14 years, and monthly regional unemployment rate, along with indicator variables for calendar months, a linear time trend, an indicator variable for the policy period, and the interaction of time trend, policy period, and household education, assets, or indicator variable for households with children > 14 years. Pairwise comparisons were made for each level of education, household assets and indicator variable for households with children > 14 years. The *p*-value is comparing the differences between the predicted values and the predicted counterfactual. The p-interaction is from Wald tests of the equality of the interaction for policy period, linear time trend, and household education, assets, or indicator variable for households with children > 14 years. Purchase data provided by Kantar WorldPanel Chile.

### Sensitivity analyses

When we conducted the analyses using the lower sweetness limits for NNS, the results remained consistent with the primary adjusted analysis (Supplementary Table 8). Compared with our primary analyses, in which NNS sweetness increased by 2.7 sucrose equivalents/capita/day, the lower sweetness limits led to an increase of 2.0 sucrose equivalents/capita/day (95% CI 1.6–2.3, *p* < 0.01). In our primary analyses, the total sweetness increased by 0.2 sucrose equivalents/capita/day. The lower sweetness limits led to a decrease of −0.6 sucrose equivalents/capita/day in total sweetness (95% CI −1.3 to 0.1, *p* = 0.12). Our results differed from the primary analyses when we conducted the analyses using the higher sweetness limits for NNS. Using the higher sweetness limits, we found a significant increase of 3.4 sucrose equivalents/capita/day in NNS sweetness (95% CI 2.9–4.0, *p* < 0.01), and the total sweetness increased by 0.9 sucrose equivalents/capita/day (95% CI 0.1–1.7, *p* = 0.03).

## Discussion

We found no significant changes in total sweetness in beverages purchased among urban Chileans compared to a counterfactual scenario if the first phase of Chile’s Food Labeling and Advertising Law was not implemented. This occurred because while there was a decrease in sugar sweetness, there was also a significant increase in the NNS sweetness compared to counterfactuals. These results suggest that there could be a compensation between the changes in NNS and total sugars sweetness because the absolute changes in sucrose equivalents in these two indices cancel each other out.

It is a positive finding that the total sweetness of household purchases did not increase after the law, as expected given the increase in NNS use in the food supply. On the other hand, it is concerning that the total sweetness of purchases did not improve. In other words, Chilean households still purchase high levels of sweet drinks irrespective of the sweetener used (sugars or NNS). This is concerning, given that multiple public health organizations have recommended reducing the intake of sweet beverages (11–14, 39–41). The main rationale behind this recommendation is that the decrease in the consumption of sweet products will lead to reductions in sugars and caloric intake. Nevertheless, the evidence on whether reduced sweetness leads to reduced sugars and calories is inconclusive (39, 42). Given that the health implications of consuming sweet beverages are still unclear, future research should continue monitoring how the consumption of sweet beverages is related to dietary intake and other health outcomes.

Our findings on NNS and total sugar sweetness align with the results from prior studies evaluating the reformulation of products in Chile after implementing the law. These studies have shown that total sugars decreased while NNS use increased after the law in beverages and dairy beverages (3, 6). Additionally, reformulated products that reduced their sugars content below the law’s cutoff were more likely to add an NNS in the post-law period (6). So far, we do not know how consumer preferences in terms of sweetness and flavors could lead to this reformulation. However, insights from a study focused on the sensory evaluation of orange juice sweetened with sugars or different NNS types showed that the juices sweetened with sugars or sucralose had greater acceptability than juices sweetened with other NNS in a small sample of college students (43).

Our results are in line with those from previous studies evaluating the changes in dietary intake and purchases after Chile’s law. When looking at dietary intake in a sample of preschoolers, a previous study showed that NNS intake increased, particularly for acesulfame K, aspartame, sucralose, and steviol glycosides (20). When analyzing purchases, prior research has found that, compared to a counterfactual, the volume purchased of regulated beverages (i.e., beverages with added sugars that surpass the law’s cutoff) and the total sugars obtained from regulated beverages decreased (23, 24). Our results show that, in beverages purchased, the sweetness from NNS increased while sweetness from total sugars decreased.

To date, our study is the first to show how the *sweetness* of beverages changed after the law and evaluate if the sweetness changed differently depending on the sweetener used (sugars or NNS). Our findings, in combination with prior studies, show that while the purchases of regulated beverages with added sugars fell (23, 24), the overall sweetness of beverages purchased remained the same after the law. Additionally, our study shows that total sugars are still the predominant source of sweetness in Chilean beverage purchases, accounting for 59% of the total sweetness.

We did not find differences in any sweetness indices by household education, households’ assets, or between households with or without children under 14 years after implementing the law. These results could indicate that the law’s implementation is not leading to differential changes in the sweetness of beverage purchases. However, it is hard to understand what happened in children because the purchase data are aggregated at the household level (i.e., we cannot see what parts of a household’s purchases are for products for children vs. adults in the same household). Future research should explore how the quantified sweetness of the dietary intake changed specifically among children after the law because they are a group of concern in which sweetened beverage intake is discouraged (12–14).

We focused on the changes in the sweetness of beverages purchased because prior studies have shown that most of the changes in total sugars and NNS reformulation, purchases, and dietary intake have been observed in beverages after the initial implementation of the law. Furthermore, beverages were the main source of sweetness in Chilean toddlers (22). However, future studies should analyze how the sweetness of foods and beverages changed after the second and third phases of the Chilean law. Foods are particularly more likely to be reformulated in these subsequent phases because the sugar threshold became stricter for solids and the limits decreased from 22.5 to 10 g of total sugars per 100 g of food.

Our study has several limitations. First, it is possible that our analyses underestimated sweetness because we did not consider synergies among NNS and, we did not consider alcohol sugars. Synergies occur when the sweetness from mixtures of sweeteners is greater than what would have been expected from each sweetener by itself (44, 45). In particular, there is a known synergy between acesulfame K and aspartame, acesulfame K and rebaudioside A (a steviol glycoside), and sucralose and rebaudioside A (45). Acesulfame K and Aspartame were commonly found in beverages, and beverages were the main dietary source of these two NNS in Chilean children before and after the law (21). Sucralose and steviol glycosides intake increased after the law, and beverages became an important dietary source of these two NNS after the law (20). The synergy between NNS allows the use of lower amounts of NNS while increasing their sweetening power, which could have led to underestimating the changes in overall sweetness. Future research focused on consumers should explore changes of sweetness perceptions to complement our findings. Additionally, we are unable to incorporate in our sweetness indices the sweetness of alcohol sugars because their content is unavailable in the Chilean NFP. Alcohol sugars are also used as sugar substitutes, and they have lower sweetness intensities than sucrose (46, 47). It is also important to note that we used observational data to examine the changes in the sweetness of beverage purchases after implementing the Chilean law, preventing us from making any causal conclusions.

This study’s main strength was the creation of the sweetness indices to measure changes in quantified sweetness. For this, we used the Chilean NFP, which provided the amounts of total sugars and NNS available in packaged beverages. In particular, the content of NNS is commonly unavailable in other countries because they are usually not declared on packaged products. We did not use data on the sweetness intensity of beverages measured by a sensory panel because there are no Chilean beverages sweetness databases. Additionally, prior studies have used international databases, and the sweetness intensity value of beverages was assigned regardless of the type of sweetener used. Our approach allowed us to estimate the total sweetness and the sweetness obtained from different sweeteners (sugars and NNS). Another strength was using a pre-post quasi-experimental modeling approach to determine the potential changes in sweetness after the law. We constructed a counterfactual (a hypothetical scenario) to understand what the post-law purchases may have looked like if the policy had not been implemented, allowing us to control secular trends.

After implementing the first phase of the Chilean Food Labeling and Advertising Law, we found that Chilean consumers are still exposed to similar sweetness levels in their beverages. Future research should explore what happened in foods after implementing the second and third phases of the law because sugar limits became stricter for solids. While the goal of the Chilean law was to reduce the purchases and intake of beverages containing high levels of sugar, it is still unknown what the implications of sustaining sweetness will be for taste preferences and population health particularly as new additives and ingredients are introduced into the packaged food and beverage supply. Countries implementing similar policies such as the one in Chile has considered the addition of a warning message regarding NNS presence; thus, it might be relevant to observe how this will impact sweetness of the food supply. Continued monitoring of these trends alongside epidemiological assessments are needed.

## Data availability statement

The original contributions presented in the study are included in the article/Supplementary material, further inquiries can be directed to the corresponding author/s.

## Ethics statement

The studies involving human participants were reviewed and approved by the Ethics Committee of the Institute of Nutrition and Food Technology of the University of Chile. Written informed consent was not provided because the data was purchased from Kantar Worldpanel Chile.

## Author contributions

NR, MB, SWN, and LST designed the research and wrote the manuscript. NR conducted the research and analyzed the data. CC provided essential materials. All authors had primary responsibility for the final content, read, and approved the final manuscript.

## Footnotes

1
https://osf.io/zmkgx
