# Supplementary material for: Did the sweetness of beverages change with the Chilean Food Labeling and Marketing Law? A before and after study
Source: Front Nutr. 2022 Oct 28;9:1043665. doi: 10.3389/fnut.2022.1043665 (PMC9650246; doi:10.3389/fnut.2022.1043665)
Supplement: Supplementary file 1 [file Data_Sheet_1.docx]

Supplementary Material

# Supplementary Figures and Tables

## Supplementary Table 1. Nutrient thresholds and implementation dates of the three stages of the Chilean Law of Food Labeling and Advertising.

| **Type of food** | **Nutrients** | **26 June 2016** | **26 June 2018** | **26 June 2019** |
| --- | --- | --- | --- | --- |
| Solids | Energy (kcal/100 g) | 350 | 300 | 275 |
|  | Total sugars (g/100 g) | 22.5 | 15 | 10 |
|  | Saturated fats (g/100 g) | 6 | 5 | 4 |
|  | Sodium (mg/100 g) | 800 | 500 | 400 |
| Liquids | Energy (kcal/100 ml) | 100 | 80 | 70 |
|  | Total sugars (g/100 ml) | 6 | 5 | 5 |
|  | Saturated fats (g/100 ml) | 3 | 3 | 3 |
|  | Sodium (mg/100 ml) | 100 | 100 | 100 |

## Supplementary Table 2. Beverages groups.

| **Food groups** | **Description** |
| --- | --- |
| Water | All water-based beverages, including flavored water and plain water, ready-to-drink, concentrate, powder, with or without added sugar; with no or <=10%m/m added juice, not carbonated |
| Sports and Energy Drinks | All sports and energy drinks, with electrolytes, hypertonic or isotonic, may have added vitamins/minerals, may have caffeine, amino acids, or herb extracts |
| Sodas | All carbonated, non-alcoholic beverages with or without added sugar, with no or <10%m/m added juice |
| Industrialized Fruit and Vegetable Juice | All beverages with fruit or vegetable (pulp, concentrate, juice) and other added ingredients; May contain sugar or sweetener or honey, water, and other additives (includes nectars, excludes 100% fruit juice) |
| 100% Fruit/Vegetable Juice | All 100% Fruit/Vegetable juice containing no added ingredients |
| Coffee and Tea | All coffee beverages, including instant, powder or granules, roasted, ground, also includes coffee substitutes of plant origin (soy, chicory, barley, other); All forms of regular and herbal teas, made from leaves or other plant parts, all forms including leaves, ground, prepared tea bags, to brew or ready-to-drink or powder. |
| Dairy-based Beverages and Dairy Substitutes | All dairy based beverages and dairy substitutes, including milk (whole, low fat, skim), dairy drinks (bebidas lacteas), drinkable yogurt, fermented milk, soy beverages, other dairy substitutes, and milk modifiers |

## Supplementary Table 3. Sucrose equivalents of Nonnutritive Sweeteners.

| **Nonnutritive sweetener** | **Compared to sucrose (table sugar)** | | | **Values used** | | |
| --- | --- | --- | --- | --- | --- | --- |
|  | **FDA(1)** | **O'Donnell(2-6)** | **Mortensen(7)** | **Mean sucrose** | **Lower limit** | **Higher limit** |
| Acesulfame K | 200x | 130-200x | 200x | 165 | 130 | 200 |
| Aspartame | 200x | 180-200x | 180-200x | 190 | 180 | 200 |
| Saccharin | 200-700x | 300-500x | 300-500x | 400 | 200 | 700 |
| Sucralose | 600x | 400-800x | 600x | 600 | 400 | 800 |
| Steviol glycoside | 200-400x | 300x | N/A | 300 | 200 | 400 |
| Cyclamate | N/A | 30-50x | 30x | 40 | 30 | 50 |

N/A: Not available

## Supplementary Table 4. Unadjusted mean sweetness of beverage purchases before and after policy implementation.

|  | **Sucrose equivalents** | | **Absolute difference** | **Relative difference** | **p-value** |
| --- | --- | --- | --- | --- | --- |
|  | **Before** | **After** |  |  |  |
| NNS^1^ sweetness | 9.1 | 10.3 | 1.2 | 12.8 | <0.01 |
| Total sugars sweetness | 19.2 | 14.8 | -4.4 | -22.9 | <0.01 |
| Total sweetness | 28.3 | 25.1 | -3.2 | -11.3 | <0.01 |

Estimates derived from OLS regressions on a post-policy period indicator variable with standard errors clustered at the household level to account for repeated measures. Purchase data obtained from Kantar WorldPanel Chile.

^1^ NNS = Nonnutritive sweeteners

## Supplementary Table 5. Unadjusted distribution of sweetness of beverage purchases before and after policy implementation.

|  | **Law period** | **Percentiles** | | | | | **p-value*** |
| --- | --- | --- | --- | --- | --- | --- | --- |
|  |  | **10** | **25** | **50** | **75** | **90** |  |
| NNS^1^ sweetness | Before | 0.0 | 1.8 | 5.7 | 12.2 | 21.8 | <0.01 |
|  | After | 0.7 | 2.8 | 6.9 | 13.6 | 22.9 |  |
| Total sugars sweetness | Before | 4.1 | 7.8 | 14.5 | 24.4 | 38.2 | <0.01 |
|  | After | 2.9 | 5.7 | 10.9 | 19.2 | 30.6 |  |
| Total sweetness | Before | 7.3 | 13.1 | 22.5 | 36.4 | 55.0 | <0.01 |
|  | After | 6.2 | 11.3 | 19.9 | 32.5 | 49.6 |  |

*P-value obtained from Kolmogorov-Smirnov test to compare differences between distributions of sweetness indices before and after the law. Purchase data obtained from Kantar WorldPanel Chile.

^1^ NNS = Nonnutritive sweeteners

## Supplementary Table 6. Mean differences between the Predicted adjusted post-policy and estimated adjusted counterfactual post-policy purchases for the sweetness of beverage purchases.

|  | **Sucrose equivalents** | | **Absolute difference (95% CI)**^1^ | **p-value** | **Relative difference** |
| --- | --- | --- | --- | --- | --- |
|  | **Counterfactual** | **Predicted** |  |  |  |
| NNS^2^ sweetness | 7.6 | 10.3 | 2.7 (2.3, 3.2) | <0.01 | 35.4 |
| Total sugars sweetness | 17.3 | 14.8 | -2.5 (-3.1, -1.9) | <0.01 | -14.6 |
| Total sweetness | 25.0 | 25.1 | 0.2 (-0.6, 0.9) | 0.68 | 0.6 |

Estimates derived from fixed-effects models comparing post-policy sweetness of beverages purchases to counterfactual post-policy sweetness of beverages purchased based on pre-policy trends for each sweetness index. Purchase data obtained from Kantar WorldPanel Chile

# ^1^ 95% CI = 95% Confidence interval

# ^2^ NNS = Nonnutritive sweeteners

## Supplementary Table 7. Mean differences between the estimated adjusted post-policy sweetness purchased from beverages and estimated adjusted counterfactual post-policy purchases by educational level, household assets, and households with or without children under 14 years.

|  | **Nonnutritive sweetener sweetness**  **(sucrose equivalents)** | | | **Total sugar sweetness**  **(sucrose equivalents)** | | | **Total sweetness**  **(sucrose equivalents)** | | |
| --- | --- | --- | --- | --- | --- | --- | --- | --- | --- |
|  | **Counterfactual** | **Predicted** | **Absolute difference** | **Counterfactual** | **Predicted** | **Absolute difference** | **Counterfactual** | **Predicted** | **Absolute difference** |
| **Education** |  |  |  |  |  |  |  |  |  |
| Less than high school | 6.7 | 9.4 | 2.8 | 17.7 | 15.6 | -2.2 | 24.4 | 25.0 | 0.6 |
| High school | 7.5 | 9.9 | 2.4 | 16.8 | 14.8 | -2.0 | 24.3 | 24.7 | 0.4 |
| College or greater | 8.6 | 11.7 | 3.1 | 17.3 | 14.0 | -3.3 | 25.9 | 25.8 | -0.2 |
| **Household assets** |  |  |  |  |  |  |  |  |  |
| Low | 6.9 | 9.3 | 2.4 | 18.2 | 15.4 | -2.7 | 25.1 | 24.8 | -0.3 |
| Middle | 7.4 | 10.0 | 2.6 | 17.1 | 14.9 | -2.2 | 24.5 | 24.9 | 0.4 |
| High | 8.7 | 11.8 | 3.1 | 16.3 | 14.0 | -2.3 | 25.0 | 25.8 | 0.8 |
| **Household with children <14 years** |  |  |  |  |  |  |  |  |  |
| No | 8.8 | 12.5 | 3.7 | 19.4 | 16.8 | -2.7 | 28.3 | 29.3 | 1.0 |
| Yes | 6.6 | 8.6 | 2.1 | 15.4 | 13.2 | -2.2 | 22.0 | 21.8 | -0.1 |

Estimates derived from fixed-effects models comparing post-policy sweetness of beverages purchases to counterfactual post-policy sweetness of beverages purchased based on pre-policy trends for each sweetness index. Covariates included head of household education level, household composition, household assets, indicator variable for households with children >14 years, and monthly regional unemployment rate, along with indicator variables for calendar months, a linear time trend, an indicator variable for the policy period, and the interaction of time trend, policy period, and household education, assets, or indicator variable for households with children >14 years. Purchase data provided by Kantar WorldPanel Chile.

## Supplementary Table 8. Results from sensitivity analyses using the lower and higher limits for sucrose equivalents.

|  | **Sucrose equivalents** | | | **p-value** | **Relative difference** |
| --- | --- | --- | --- | --- | --- |
|  | **Counterfactual** | **Predicted** | **Absolute difference (95% CI)**^1^ |  |  |
| **NNS^2^ sweetness** |  |  |  |  |  |
| Lower limit | 6.2 | 8.2 | 2.0  (1.6, 2.3) | <0.01 | 31.6 |
| Higher limit | 9.1 | 12.6 | 3.4  (2.9, 4.0) | <0.01 | 37.8 |
| **Total sweetness** |  |  |  |  |  |
| Lower limit | 23.5 | 23.0 | -0.6  (-1.3, 0.1) | 0.12 | -2.4 |
| Higher limit | 26.4 | 27.4 | 0.9  (0.1, 1.7) | 0.03 | 3.4 |

Estimates derived from fixed-effects models comparing post-policy sweetness of beverages purchases to counterfactual post-policy sweetness of beverages purchased based on pre-policy trends for each sweetness index. Purchase data obtained from Kantar WorldPanel Chile

^1^ 95% CI = 95% Confidence interval

^2^ NNS = Nonnutritive sweeteners
